# FFLAME: a fragment-to-framework learning approach for MOF potentials

**DOI:** 10.1039/d5dd00321k

**Published:** 2025-10-30

**Authors:** Xiaoqi Zhang, Yutao Li, Xin Jin, Berend Smit

**Affiliations:** a Laboratory of Molecular Simulation (LSMO), Institut des Sciences et Ingénierie Chimiques, École Polytechnique Fédérale de Lausanne (EPFL) Rue de l'Industrie 17 CH-1951 Sion Switzerland berend.smit@epfl.ch

## Abstract

Metal–organic frameworks (MOFs) exhibit immense structural diversity and hold promise for applications ranging from gas storage and separation to energy storage and conversion. However, structural flexibility makes accurate and scalable property prediction difficult. While machine learning potentials (MLPs) offer a compelling balance between accuracy and efficiency, most existing models are system-specific and lack transferability across different MOFs. In this work, we introduce FFLAME – Fragment-to-Framework Learning Approach for MOF Potentials, a fragment-centric strategy for training transferable MLPs. By decomposing MOFs into their constituent metal clusters and organic linkers, FFLAME enables efficient reuse of chemical environments and significantly reduces the need for full-framework training data. We demonstrate that fragment-informed training improves model generalizability, particularly in data-scarce regimes, and accelerates convergence during fine-tuning. FFLAME achieves near-target accuracy on unseen MOFs with minimal additional training. These results establish a robust and data-efficient pathway toward general-purpose MLPs for the simulation of diverse framework materials.

## Abstract 2

FFLAME, a fragment-centric strategy for training transferable MOF machine learning potentials, learns from building blocks, lowers data needs, and achieves near-target accuracy with minimal fine-tuning even for unseen MOFs.

## Graphical abstract

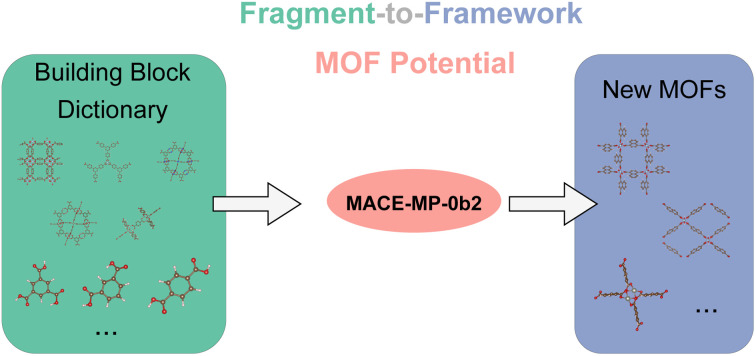

## 1 Introduction

Metal–organic frameworks (MOFs) have attracted considerable attention due to their potential in a wide range of applications, including carbon capture,^1,2^ hydrogen and methane storage,^3–5^ gas separation,^6,7^ and water vapor adsorption.^8^ This versatility stems from their modular architecture, high surface areas, and highly tunable pore structures. MOFs consist of metal nodes and organic linkers that self-assemble into extended reticular frameworks, giving rise to an expansive chemical design space.^9^ However, identifying optimal MOFs for specific applications remains a major challenge due to the enormous diversity of possible structures.^10^

The performance of MOFs is strongly influenced by their physical properties and chemical characteristics, including mechanical and thermal stability,^11–13^ thermal conductivity,^14,15^ and heat capacity.^16,17^ Among these, Witman *et al.*^18^ further demonstrated that the structural flexibility of MOFs significantly influences their performance in gas separation applications. Classical force-field-based approaches, such as UFF4MOF,^19,20^ DREIDING,^21^ and BTW-FF,^22^ enable high-throughput screening. However, they often lack the precision required to capture subtle yet critical phenomena and can generate unphysical configurations, such as distorted aromatic rings. Properties that are sensitive to vibrational modes, such as negative thermal expansion coefficients, breathing behavior of MIL-53, and polar gas adsorption in certain classes of MOFs,^23^ require more sophisticated interaction models.^24^ While density functional theory (DFT) provides high-accuracy predictions of these properties, its computational cost is prohibitive for large-scale screening and dynamic simulations.

In this context, machine learning potentials (MLPs) present a promising middle ground, combining near-DFT accuracy with substantially reduced computational cost.^25,26^ By learning from quantum data, MLPs facilitate realistic simulations of MOF structures, energetics, and dynamics at scale.^27^ Recent studies have demonstrated the effectiveness of MLPs trained on individual, well-characterized MOFs. For instance, Vandenhaute *et al.*^28^ developed an MLP for MIL-53 that successfully predicted its phase transition pressure. Similarly, Wieser and Zojer^27^ computed the thermal conductivity of MOF-5 using a machine-learned force field in agreement with single-crystal experimental values. However, such models are typically system-specific and lack transferability. Extending these approaches to new MOFs often necessitates generating extensive new training data and retraining from scratch, thereby limiting their scalability and broader applicability.

To address this, recent efforts have focused on developing more general and transferable MLPs for MOFs. One straightforward strategy involves training models on large and diverse datasets of MOFs. For example, Yue *et al.*^29^ trained an MLP on approximately 3000 Zn-based MOFs. Alternatively, transfer learning from a pre-trained foundation model can improve data efficiency. Elena *et al.*,^30^ for instance, fine-tuned the MACE model using 127 carefully selected MOFs. Although MACE offers a reasonable starting point, it was initially trained on the MPtrj dataset of 150 000 inorganic crystals,^31,32^ whose chemical environments differ substantially from those in MOFs. As a result, even with fine-tuning, the resulting models exhibit limited generalization beyond their training domain, and it remains unclear which classes of MOFs they can reliably extrapolate to. This underscores a core challenge in the field: current models often lack both interpretability and systematic transferability to previously unseen frameworks.

A natural next step is to incorporate organic covalent bonding environments into the refinement of MACE. A related strategy, training machine learning potentials for MOFs using atomic clusters, has been proposed to bypass the high cost of *ab initio* calculations of bulk materials,^33^ particularly in challenging cases involving open metal sites^34^ or defects.^35^ However, the transferability of such cluster-based models to other materials sharing similar structural building blocks remains to be explored. Rather than relying on the careful selection of chemically inequivalent atoms to define representative clusters of a bulk structure, we instead leverage the intrinsic modularity of MOFs by decomposing them directly into their building blocks. This fragment-centric view forms the basis of the framework we develop in this work.

In this work, we introduce FFLAME – Fragment-to-Framework Learning Approach for MOF Potentials, a strategy for building transferable MLPs for MOFs using a fragment-centric representation. By decomposing MOFs into metal clusters and organic linkers, we train models that can generalize across frameworks assembled from these constituent fragments. This strategy enables efficient reuse of learned chemical environments, substantially reducing the data required to model new MOF structures with high accuracy. Importantly, even when a target MOF is excluded from the training set, FFLAME can achieve near-target accuracy if its building blocks are included, often requiring only minimal fine-tuning.

## 2 Outline of the methodology

A detailed description of the computational methods used is provided in Section 5. Here, we describe the system we study and provide evidence that the methodology leads to a more efficient route for developing a machine learning potential.

### 2.1 Selection of the building blocks

**Fig. 1:**
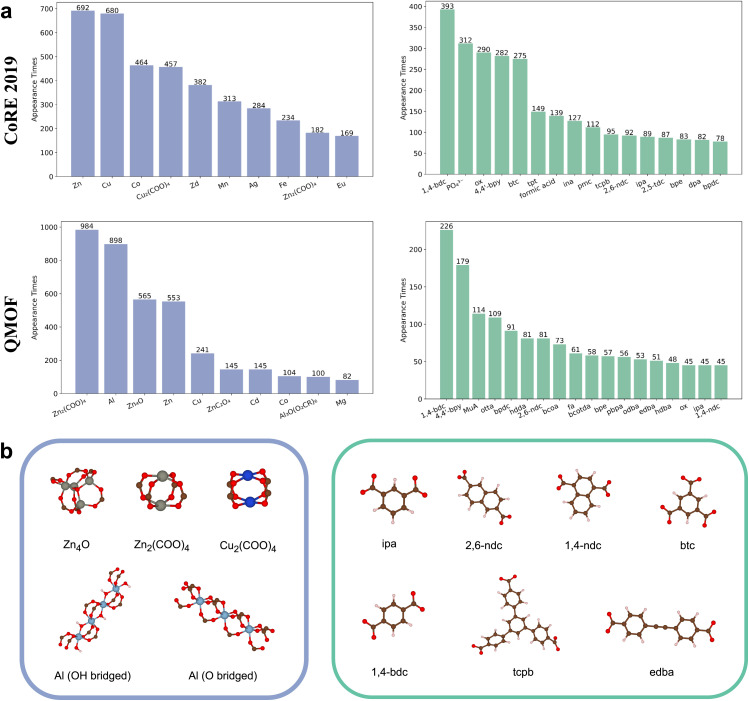
(a) Statistical distribution of building blocks in the CoRE 2019 and QMOF datasets. The most common metal nodes and organic ligands show notable overlap between the two datasets. (b) Molecular structures of the five selected metal nodes and seven selected ligands used to construct the building block library.

To construct a representative library of MOF building blocks, we analyzed structures from the CoRE 2019 ^36^ and QMOF^37,38^ databases by decomposing them into metal nodes and organic linkers using MOFfragmentor.^39^Fig. 1a summarizes the most frequently occurring building blocks in the two datasets.

Among metal nodes, Zn and Cu are the most prevalent elements, consistently appearing across both databases. The CoRE 2019 dataset, in particular, exhibits a broader diversity in metal node chemistry, likely due to the experimental origins of its structures.

For organic ligands, common examples include 1,4-benzenedicarboxylic acid (1,4-bdc), 4,4′-bipyridine (4,4′-bpy), benzene-1,3,5-tricarboxylic acid (btc), *etc.* The structures corresponding to the ligand abbreviations are provided in the SI. Notably, some less conventional ligands, such as phosphoric acid (H_3_PO_4_), formic acid, and oxalic acid (ox), also appear frequently in the CoRE 2019 database. These species are likely used as structural modulators during the synthesis of MOFs.

Based on the statistical distributions observed, we focus on three metal species, Zn, Cu, and Al. We further excluded structures with extra elements other than C, H, and O. In the rest of the frequently occurring building blocks, we selected five metal nodes and seven organic ligands, aiming to maximize the diversity of their possible combinations. These selected building blocks establish a basic building block library for training a general-purpose machine learning potential for MOFs, which are illustrated in Fig. 1b. A total of 25 MOFs composed of these chosen building blocks were identified within the two databases and used for subsequent modeling and analysis in Section 3.

### 2.2 Proof of concept

To evaluate the impact of incorporating building block configurations during machine learning potential training, we conduct experiments on three MOFs: CAU-10-OCH_3_, Zn_4_O(TCPB)_*n*_, and MOF-14. These MOFs feature distinct metal node motifs: Al chain bridged by O, Zn_4_O, and a Cu paddlewheel, respectively. We fine-tune the MACE-MP-0b2 model (hereafter referred to as MACE) for each MOF, both with and without the inclusion of building block configurations. Discussion on the selection of the MACE foundation model is provided in the SI.

To sample ligand configurations, we begin by extracting them from MOF structures. Molecular dynamics (MD) simulations and geometry optimizations are first performed on the MOFs. Details can be found in Section 5. Ligand configurations are then extracted from the MOF trajectories using the MOFfragmentor package.^39^ These extracted ligands lack hydrogen atoms at the metal coordination sites. Missing hydrogens are added using functions from MOFChecker,^40^ and their positions are subsequently optimized using the xTB force field.^41^ A total of 500 representative ligand configurations are selected *via K*-means clustering.^42^

In contrast to ligands, directly isolating physically realistic metal node structures from MOFs is more challenging. To address this, we identify alternative MOFs that contain the same metal node but differ in their ligands. MD trajectories from these MOFs are used to sample 50 representative metal node configurations.

We refer to the MOF of interest as the target MOF. Configurations of the target MOF are sampled from the same MD trajectories used for ligand extraction. Based on these data, we construct four types of training sets: (1) configurations of the target MOF alone (referred to as frameworks), (2) frameworks and metal node configurations, (3) frameworks and ligand configurations, and (4) frameworks, metal nodes, and ligands. Excluding the training configurations, the test set was sampled from the remaining configurations in the MD trajectories. It consists exclusively of the target MOF configurations. All four types of training sets share the same test set.

**Fig. 2:**
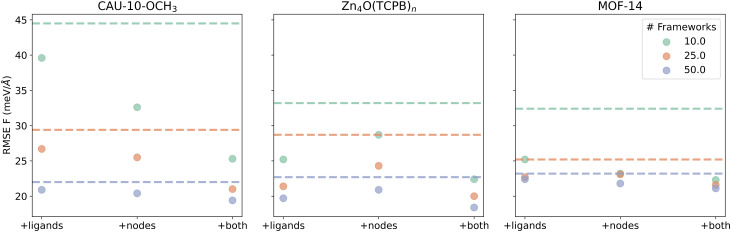
Model performance of fine-tuning MACE with and without building blocks. Dashed lines indicate baseline models fine-tuned using only framework configurations. Adding ligand and/or metal node configurations improves model accuracy and allows comparable or better performance using fewer framework configurations.

Since all test-set energy errors after fine-tuning are below 1.5 meV per atom, and the inclusion of building blocks has negligible impact on these energy errors, we focus on force errors to evaluate model performance, as shown in Fig. 2. Models fine-tuned using only target MOF configurations serve as benchmarks and are indicated by dashed lines. Different colors represent varying numbers of target MOF configurations in the training set.

In all three cases, supplementing the training set with either ligand or metal node configurations reduces the force errors compared to the framework-only baseline. Incorporating both leads to further improvements. However, the gains show diminishing returns: as the number of framework configurations increases, the added benefit from building blocks becomes less pronounced.

Notably, a model fine-tuned with 10 target MOF configurations—augmented with building block data—can match or even outperform one fine-tuned on 50 framework configurations alone. This suggests that incorporating ligand and metal node configurations enriches the diversity of local atomic environments, thereby enhancing the model's ability to generalize from fewer framework samples. This strategy offers an efficient and accurate approach for developing a fundamental and transferable MLP for MOFs.

### 2.3 Ligand sampling method

The approach for obtaining ligand configurations described above is to extract ligands from MD simulations of MOFs containing the ligands, which are referred to as “constrained” ligands. An alternative method is to run molecular simulations on isolated ligands. The ligand configurations obtained by this method are referred to as “free” ligands. We applied both strategies to the seven ligands selected in Section 2.1, obtaining two sets of training and test datasets.

**Fig. 3:**
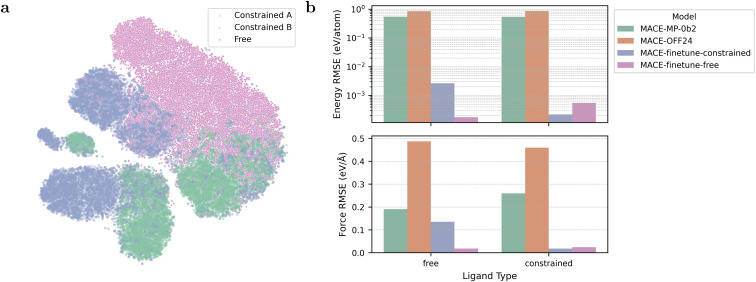
(a) Visualization of edba ligand configurations from three sources: extracted from MOF A, extracted from MOF B, and sampled from MD simulations of the isolated ligand. The configuration space sampled from the isolated ligand broadly covers that of the constrained configurations. (b) Comparison of model performance on free and constrained ligand test sets. The model fine-tuned with the free ligand training set performs well on both test sets, demonstrating strong generalization.

In Fig. 3a, we visualize the configuration spaces sampled by the two methods for a flexible ligand (edba) among the selected ligands in the two-dimensional space. The constrained configurations are extracted from two MOFs that contain this ligand, which are labeled as “Constrained A” and “Constrained B”. As shown in Fig. 3a, the configuration space of free ligands covers the major part of the constrained ligand spaces, although the distributions differ.

It is interesting to compare our fine-tuned MACE models (MACE-finetune-free and MACE-finetune-constrained) with the original MACE model (MACE-MP-0b2) and MACE-OFF24. MACE-finetune-free is fine-tuned using the free ligand training dataset, while MACE-finetune-constrained is fine-tuned using the constrained ligand training dataset. MACE-OFF24 is a machine learning force field specifically developed for organic molecules.

The result of this comparison is shown Fig. 3b. Surprisingly, MACE-OFF24 exhibited higher errors than MACE-MP-0b2 in both the energy and force evaluations. Our fine-tuned models achieved lower energy and force errors than the original MACE model. Notably, the model fine-tuned with free ligand data performed well on both free and constrained ligand test sets, demonstrating strong generalization. In contrast, the model trained on constrained ligands failed to predict the configurations of the free ligands accurately. Based on these results, we adopt the free ligand sampling method for constructing ligand configuration datasets in the remainder of this study.

## 3 The FFLAME workflow

The aim of FFLAME is to develop a workflow that allows us to systematically create a machine learning force field sufficiently accurate to simulate MOFs out of the box, or in some cases with minimal fine-tuning.

### 3.1 Description of the workflow

**Fig. 4:**
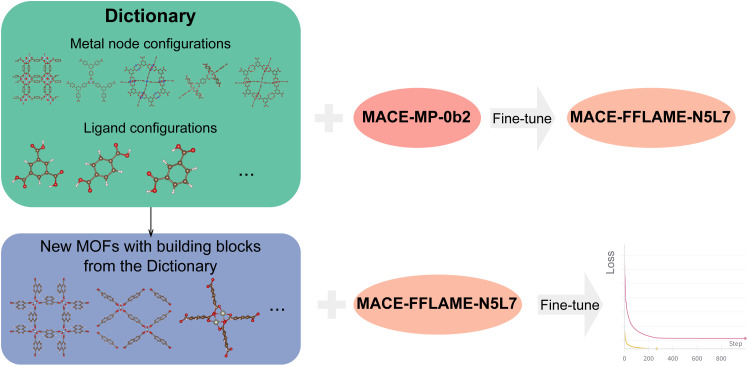
Workflow of FFLAME. Selected metal nodes and ligands act as fundamental building blocks (“words”) forming a shared “dictionary” for MOF representation. By fine-tuning the MACE model on configurations of metal nodes (MOF-derived) and isolated ligands, a general-purpose model, MACE-FFLAME-N5L7, is obtained. This model serves as a more efficient and accurate starting point for adapting to new MOFs composed of known building blocks.

The workflow of FFLAME is illustrated in Fig. 4. In this approach, selected metal nodes and ligands are treated as “words”, collectively forming a “dictionary” that underpins the construction of a fundamental machine learning potential for MOFs. By fine-tuning the MACE model on this curated dictionary, we obtain a transferable potential, MACE-FFLAME-N5L7, where N5L7 refers to the selected five metal nodes and seven organic ligands. MACE-FFLAME-N5L7 can serve as a foundation for modeling new MOFs that share components within the dictionary.

To build this fundamental model, we selected 5 MOFs from the set of 25 that featured distinct metal nodes, and performed molecular dynamics simulations to sample their metal node configurations. These were combined with the previously described free ligand training dataset and used to fine-tune the MACE model. To quantify model uncertainty, we repeated the fine-tuning process using three random seeds, generating an ensemble of models to estimate prediction deviations. Additional details on the fine-tuning procedures are provided in Section 5.

### 3.2 Performance of FFLAME

**Fig. 5:**
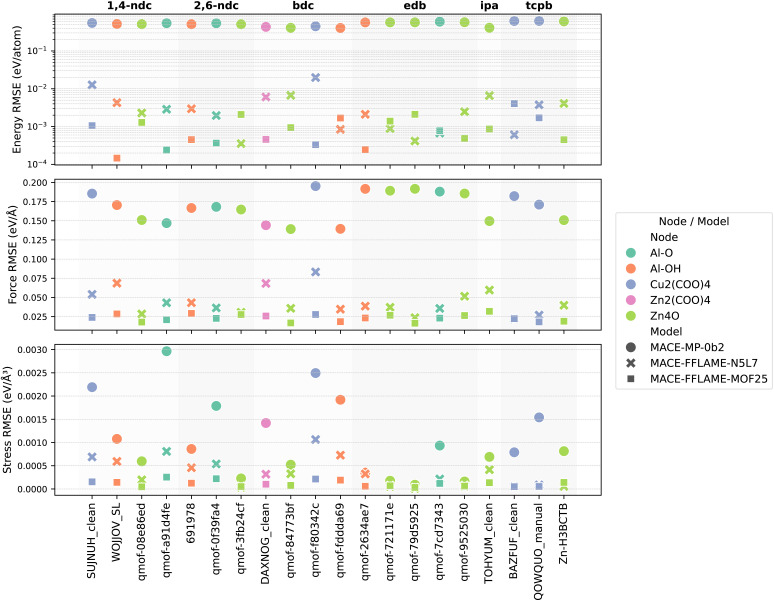
Performance of MACE-FFLAME-N5L7 and MACE-FFLAME-MOF25 on 20 MOFs. Comparison of energy (top), force (middle), and stress (bottom) errors for the original MACE model, MACE-FFLAME-N5L7, and MACE-FFLAME-MOF25. Fine-tuning MACE with a small set of metal node and ligand configurations (N5L7) significantly improves model performance on MOFs not included in the training set, while requiring substantially fewer data points than traditional framework-based training. A second round of fine-tuning, incorporating prediction deviation-guided configurations and strained structures, results in MACE-FFLAME-MOF25, a more accurate and general model across all 25 MOFs.

As shown in Fig. 5, MACE-FFLAME-N5L7 achieves significantly lower energy, force, and stress errors across the remaining 20 MOFs compared to the original MACE model, although the 20 MOFs are not in the training set. For a few structures among them, the accuracy is sufficient to perform simulations.

Next, we performed NVT and NPT simulations using MACE- FFLAME-N5L7 for the 20 MOFs, and sampled configurations with high prediction deviations. In total, 489 such configurations were collected. Additionally, six strained configurations were generated per MOF, resulting in a final set of 609 configurations. We then fine-tuned MACE-FFLAME-N5L7 using these data to obtain a more accurate and generalizable potential across all 25 MOFs (including the five MOFs used for sampling metal node configurations), which we refer to as MACE-FFLAME-MOF25.

Although energy errors slightly increased for a few MOFs in this second round of fine-tuning, all remained within acceptable limits. Importantly, this strategy contrasts with the traditional approach of training a universal MOF potential by sampling an equal number of configurations from each structure. Instead, FFLAME adaptively focuses on MOFs where the model exhibits high force prediction deviations, allowing for more targeted data acquisition and more efficient fine-tuning.

### 3.3 Transferability of FFLAME

To evaluate the transferability of our approach, we selected ten Al-based MOFs from the work of Li *et al.*,^43^ whose metal nodes and organic ligands exhibit structural similarities to those in our training dataset.

**Fig. 6:**
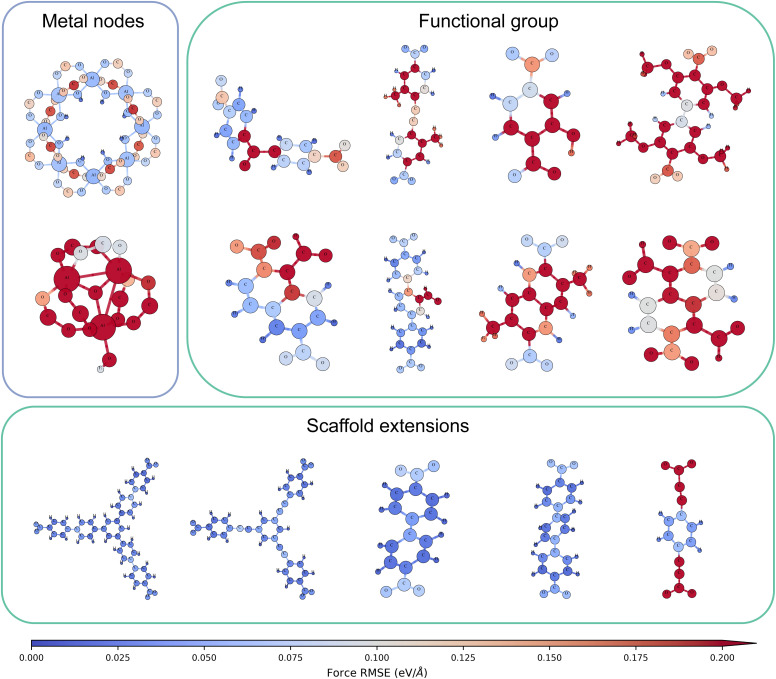
Predicted force errors of MACE-FFLAME-N5L7 on metal nodes and organic ligands structurally similar to the selected motifs. The model performs well on metal nodes with similar coordination environments. The ligands are grouped into two categories: functional group modifications and scaffold extensions. Functional group modifications generally increase force errors for the modified atoms and their neighboring atoms, while the model demonstrates excellent transferability for ligands corresponding to scaffold extensions.

To identify the sources of errors, we visualized per-atom force errors predicted by MACE-FFLAME-N5L7, as shown in Fig. 6. The force errors of MACE-FFLAME-N5L7 remain below 0.1 eV Å^−1^ in Fig. 5. Thus, in the visualization, atoms with errors below 0.1 eV Å^−1^ are depicted in blue, whereas those above this threshold are shown in red.

In Fig. 6, two types of Al-containing secondary building units are present. In the first, each Al atom is coordinated to six oxygen atoms, forming an eight-membered ring. This coordination environment, even though with a different geometry, closely resembles those in Al chain (O or OH bridged) nodes from the training set, resulting in low force errors on the Al atoms. In contrast, the second type consists of three Al atoms bridged by a single oxygen atom. Here, two Al atoms are coordinated to five oxygen atoms, while the third is coordinated to six oxygen atoms. However, the latter exhibits a bond length distinct from those encountered in the training data, leading to poor transferability of our model in this case.

The ligand modifications can be categorized into two classes: introduction of functional groups and scaffold extensions.

We evaluated the effect of functional groups, including carbonyl, aldehyde, methyl, hydroxyl, and methoxy. In general, the introduction of functional groups results in high force errors in the functional group atoms and their neighborhoods. Depending on the composition and the position of the functional groups, the influence on the rest of the atoms varies slightly. If the functional group is near the carboxyl group coordinated to the metal nodes, it can increase the force errors of the carboxyl atoms by affecting their rotational flexibility. In addition, aldehyde or hydroxyl groups may further raise errors in nearby oxygen atoms due to potential hydrogen-bonding interactions, which are absent in our model's training set.

For ligands involving scaffold extensions, the model typically transfers well. MACE-FFLAME-N5L7 shows consistently low force errors for the first four ligands in this class, as shown in Fig. 6. The last case, however, presents an exception. Although the model has encountered conjugation between alkynes and benzene rings in the training data (*e.g.*, through edba), it has not been exposed to configurations combining alkynes with carboxyl groups. Consequently, the model exhibits high force errors on atoms belonging to these motifs.

### 3.4 Application: thermal behaviors of MOFs

We computed the lattice coefficients of thermal expansion (CTEs) for three MOFs with available experimental data using our model (MACE-FFLAME-MOF25) and the model reported by Elena *et al.*^30^ The results are summarized in Table 1. Unlike their study, which employed quasi-harmonic phonon calculations, we used MD simulations and therefore obtained a different CTE from the value reported in their work (−6.65 × 10^−6^ K^−1^) for MOF-5. Overall, both models show good agreement with experiments and reliably capture the CTEs.

**Table 1:** The CTE of MOF-5, HKUST-1, and MOF-14 at 300 K, obtained from experiments and simulations. Values are given in units of 10^−6^ K^−1^

MOF	MACE-FFLAME-MOF25	Experiment	MACE-MP-MOF0-v2
MOF-5	−13.6	−13.1 (ref. 44)	−14.2
HKUST-1	−3.7	−4.1 (ref. 45)	−2.9
MOF-14	−9.7	−11 (ref. 45)	−13.1

### 3.5 Application: rotational barrier of phenylene group in MOF-5

**Fig. 7:**
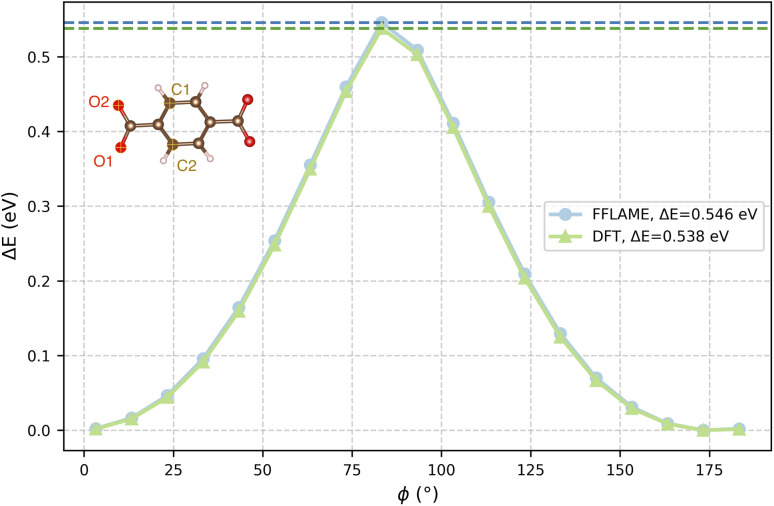
Energy difference as a function of the rotational angle of the phenylene group in MOF-5. The rotation angle *ϕ* is defined by the dihedral O1–O2–C1–C2.

To validate our model's performance for high-energy configurations, we computed the rotational energy barrier of the phenylene group in MOF-5. A series of structures with varying rotation angles is generated, and the energy of each structure is calculated using both MACE-FFLAME-N5L7 and DFT. The barrier predicted by MACE-FFLAME-N5L7 is 0.546 eV, in excellent agreement with the DFT value of 0.538 eV, as shown in Fig. 7. Our result also falls within the range of experimental measurements (0.490(87) eV)^46^ and the predictions of other machine learning potentials (0.49–0.56 eV).^33,47^ Further comparison of the predicted and DFT-calculated forces and stresses for these MOF-5 configurations is provided in the SI.

## 4 Conclusions

In this work, we present a modular strategy for training MLPs for MOFs based on their constituent building blocks. By systematically incorporating configurations of individual building blocks into the training pipeline, we demonstrate that models require significantly fewer bulk configurations to achieve accurate predictions. Our fine-tuning experiments on three representative MOFs confirm that including building block data improves model accuracy, particularly in data-scarce regimes.

Building on this foundation, we introduce FFLAME, which leverages the dictionary of building blocks to fine-tune a generalizable MOF potential. The dictionary guides the scope of new MOFs to which the model can be generalized. FFLAME exhibits excellent performance on unseen MOFs and converges with fewer fine-tuning epochs compared to models initialized from MACE (see SI for more details). This reduction in training time is especially valuable when developing general MLPs across large datasets. By examining per-atom errors on similar building blocks, we gain insights into the extent to which the fine-tuned model can be transferred.

Overall, our results highlight the efficiency and effectiveness of building block–based training strategies, offering a promising path toward accurate, transferable, and data-efficient MLPs for complex framework materials.

## 5 Methods

### 5.1 Dataset preparation

The initial training set of MOFs was generated from MD trajectories. Simulations were performed using the MACE-MP-0b2 calculator in the canonical (NVT) ensemble, employing a Langevin thermostat^48^ with a time step of 1 fs over a total duration of 50 ps. Trajectories were sampled at four temperatures: 100, 300, 500, and 600 K to capture thermally accessible configurations across a wide range of thermal conditions. Temperatures above 600 K were avoided to minimize the likelihood of generating unphysical structures.

To improve the model's ability to predict stress, additional configurations were included by applying uniform volumetric strain to the unit cells. Six strained structures were generated per MOF by systematically expanding and compressing the lattice in all directions, with volume variations ranging from −10% to +10%.

For free ligands, forces and energies were calculated using the GFN1-xTB semiempirical potential.^41^ MD simulations were run at 100, 300, 500, and 600 K. 100 representative configurations were selected from the MD trajectories for geometry optimization. The MD and optimization trajectories were combined for sampling to enrich the training dataset.

For the twenty MOFs not included in the initial training set, NPT and NVT simulations were performed using the fine-tuned fundamental model, MACE-FFLAME-N5L7, to explore more realistic structural fluctuations. These simulations were used to identify configurations where the model predictions showed large deviations. Such outlier configurations were selected for DFT single-point calculations and subsequently included in the second round of model fine-tuning.

All simulations and post-processing were conducted using the Atomic Simulation Environment (ASE), interfaced with various calculators as needed.^49^

### 5.2 *K*-Means sampling and visualization

To efficiently sample representative configurations from the molecular dynamics trajectories, atomic environments were first encoded using Smooth Overlap of Atomic Positions (SOAP) descriptors,^50^ computed with the Python package DScribe v2.1.1.^51^ We use SOAP to featurize MOFs due to its hand-crafted characteristics.

To construct training, validation, and test datasets, we applied *K*-means clustering^42^ to the SOAP feature space. The full configuration space was partitioned into *N* clusters, where *N* corresponds to the number of configurations to be selected. The configuration closest to each cluster center was selected as a representative sample. This approach ensures that the final dataset spans the structural diversity observed in the simulation trajectories while reducing redundancy.

For visualization, the SOAP features were projected into two dimensions using the *t*-distributed Stochastic Neighbor Embedding (*t*-SNE) algorithm.^52^ The implementation from the scikit-learn Python library^53^ was used, with the perplexity set to 30 and the learning rate set to auto.

### 5.3 DFT calculations

The quickstep code of the CP2K^54^ software was used to perform DFT calculations for labeling configurations. These calculations were performed using Perdew–Burke–Ernzerhof (PBE) functional within the generalized gradient approximation (GGA),^55^ with Grimme's D3 corrections for van der Waals (vdW) interactions.^56^ If the lattice parameter was smaller than 10 Å, a supercell was used. We employed the orbital transformation method with a plane-wave cutoff of 600 Ry, a relative cutoff of 50 Ry, and a self-consistent field (SCF) convergence threshold of 1 × 10^−6^. The calculations used *Γ* point sampling. The CP2K input script can be found on Zenodo.^57^

A good initial guess of the wavefunction can significantly reduce the number of iterations for SCF convergence. This strategy has already been widely used to accelerate *ab initio* molecular dynamics or geometry optimization by DFT calculation. To extend this idea to DFT labeling, we sorted configurations by structural similarity and performed DFT calculations sequentially. Except for the first configuration, the remaining configurations use the converged wavefunction of the previous one as the initial guess. To determine an optimal ordering in which adjacent configurations are globally maximally similar, each configuration was treated as a node in a fully connected graph. Similarity between configurations was defined by the Euclidean distance between their SOAP descriptors, which served as the edge weights between nodes. A minimum spanning tree (MST) was constructed to connect all nodes with the lowest possible total edge weight. A depth-first traversal of the MST was then used to generate the final sequence of configurations. Compared to performing the DFT single-point calculations for each configuration from scratch, this ordering strategy reduced the total calculation time by 48% for 100 IRMOF-8 configurations and by 22% for 100 isolated tcpb linker configurations.

### 5.4 Fine-tuning MACE

Fine-tuning was performed on the pre-trained MACE-MP-0b2 (medium) model^58^ using the MACE codebase (version 0.3.13).^31,32^ Details of the model selection can be found in the SI. The model architecture was retained from the original; it uses 128 channels with a maximum message-passing angular momentum quantum number of max_*L* = 1. Local atomic environments were defined using a cutoff radius of 5 Å, and interatomic distances were encoded using eight radial basis functions.

We applied the multihead replay fine-tuning mechanism to avoid catastrophic forgetting. A two-stage fine-tuning strategy was adopted to balance the learning of energy, force, and stress contributions. In the first stage, the loss weights were set to 1 : 10 : 100 for energy, force, and stress, respectively. In the second stage, the weights were adjusted to 10 : 1 : 1 to focus on accurate energy prediction. Optimization was performed with a batch size of 8 using an initial learning rate of 1 × 10^−3^ with a weight decay of 5 × 10^−7^. In the second stage, the learning rate was reduced to 1 × 10^−4^. Early stopping was employed with a patience parameter of 10 epochs to prevent overfitting.

### 5.5 Coefficient of thermal expansion calculation

To calculate the CTE for the selected MOFs, we performed MD simulations in the NPT ensemble over the temperature range of 100 K to 500 K, in increments of 100 K, using LAMMPS.^59^ Interatomic energies, forces, and stresses were evaluated with our fine-tuned model, MACE-FFLAME-MOF25 and MACE-MP-MOF0-v2 from the work of Elena *et al.*^30^ Each simulation was run for a total of 1 × 10^5^ steps with a timestep of 1 fs. For each temperature, the average lattice parameters were computed after an initial equilibration period of approximately 40 ps.

## Author contributions

X. Z. curated the datasets and fine-tuned the models. Y. L. conceived the project and performed the DFT calculations. X. J. checked unphysical structures. B. S. supervised the project. All authors reviewed, edited, and approved the manuscript.

## Conflicts of interest

The authors declare no competing interests.

## Supplementary Material

DD-004-D5DD00321K-s001

## Data Availability

The Supplementary Information provides addition details, including schematic structures of the ligands, a comprehensive list of structures used in this work, the rationale for selecting the MACE foundation model, extended analyses of the cases presented in the "Proof of Concept" section, parity plots illustrating model performance, and a comparison of training efficiency and convergence speed. See DOI: https://doi.org/10.1039/d5dd00321k. All data and the fine-tuned models in this work are available for download on Zenodo at https://doi.org/10.5281/zenodo.15784714.^57^ Code availability: The Python codes used to prepare training data and fine-tune the model are available at https://github.com/XiaoqZhang/MACE-FFLAME-toolkit.git.
