# Supplementary material for: FFLAME: a fragment-to-framework learning approach for MOF potentials
Source: Digit Discov. 2025 Oct 30;4(12):3466–77. doi: 10.1039/d5dd00321k (PMC12593188; doi:10.1039/d5dd00321k)
Supplement: DD-004-D5DD00321K-s001 [file DD-004-D5DD00321K-s001.pdf]

Supplementary Information (SI) for Digital Discovery.
This journal is © The Royal Society of Chemistry 2025

Supplementary Information: FFLAME: A Fragment-to-Framework Learning Approach for MOF Potentials

Xiaoqi Zhang [1], Yutao Li [1], Xin Jin [1], and Berend Smit *[1]

[1]Laboratory of molecular simulation (LSMO), Institut des Sciences et Ingénierie Chimiques, École Polytechnique Fédérale de Lausanne (EPFL), Rue de lIndustrie 17, CH-1951 Sion, Switzerland

These authors contributed equally to this work.
*Corresponding author: berend.smit@epfl.ch

Contents

1 Schematic structures of ligands 3

2 List of structures 4

3 Choice of MACE foundation model 5

4 The number of building blocks in training 6

5 Model performance on the twenty test MOFs 7

6 Model performance on the five MOFs of type II 11

7 Force and stress errors on MOF-5 12

8 Faster training convergence 13

1 Schematic structures of ligands

The schematic structures of representative organic ligands, along with their abbreviations, are shown in Fig. S1. These ligands were extracted from the CoRE 2019[1] and QMOF[2,3] databases and are used throughout the main text for consistency and clarity.

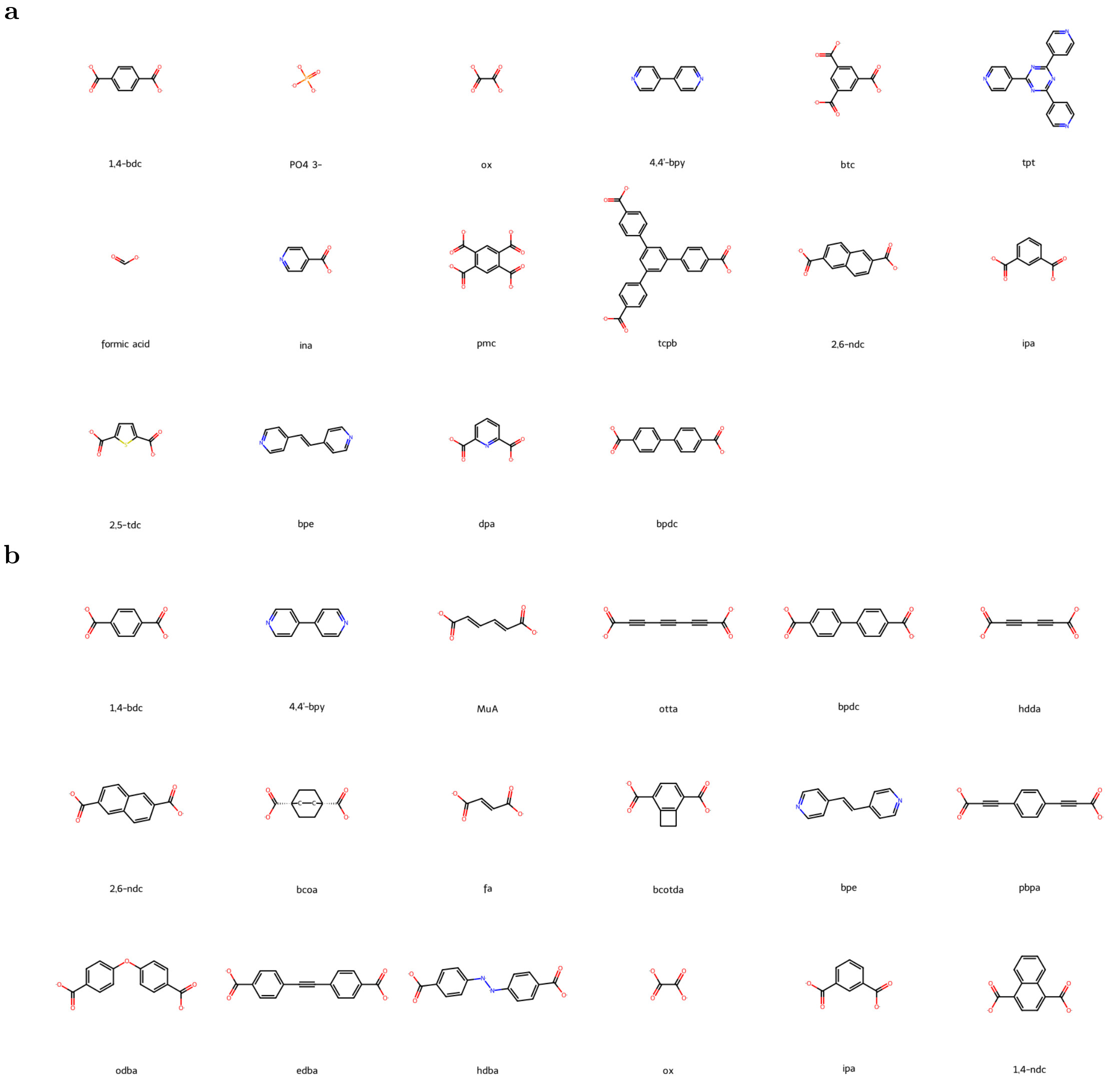

Supplementary Figure S1: Schematic structures and abbreviations of prevalent ligands found in (a) the CoRE 2019 and (b) QMOF databases.

2 List of structures

ID	CIF name	Common name	Metal node	Organic linker	Notes
a		CAU10-O-CH_3	Al-OH	5-Methoxybenzene-1,3-dicarboxylic acid	Type I: Structures used for proof-of-concept demonstration.
b		$Zn_4O(TCPB)_n$	Zn_4O	tcpb	
c		MOF-14	$Cu_2(COO)_4$	tcpb	
i	qmof-11f7d05		Al-O	tcpb	Type II: Five MOFs used for the training of MACE-FFLAME-N5L7 to provide metal node configurations.
ii	CAU-10	CAU10	Al-OH	ipa	
iii	IKETOH_manual	HKUST-1(Cu)	$Cu_2(COO)_4$	btc	
iv	RIFDUG01_clean	HKUST-1(Zn)	$Zn_2(COO)_4$	btc	
v	SAHYIK	MOF-5	Zn_4O	bdc	
1	qmof-a91d4fe		Al-O	1,4-ndc	Type III: A total of 20 MOFs consisted of the selected building blocks.
2	qmof-0f39fa4		Al-O	2,6-ndc	
3	qmof-7cd7343		Al-O	edb	
4	WOJJOV_SL		Al-OH	1,4-ndc	
5	691978		Al-OH	2,6-ndc	
6	qmof-fddda69	MIL-53	Al-OH	bdc	
7	qmof-2634ae7		Al-OH	edb	
8	SUJNUH_clean		$Cu_2(COO)_4$	1,4-ndc	
9	qmof-f80342c		$Cu_2(COO)_4$	bdc	
10	QOWQUO_manual	MOF-14	$Cu_2(COO)_4$	tcpb	
11	BAZFUF_clean	MOF-143	$Cu_2(COO)_4$	tcpb	
12	DAXNOG_clean		$Zn_2(COO)_4$	bdc	
13	qmof-08e86ed	IRMOF-7	Zn_4O	1,4-ndc	
14	qmof-3fb24cf	IRMOF-8	Zn_4O	2,6-ndc	
15	qmof-84773bf		Zn_4O	bdc	
16	qmof-9525030		Zn_4O	edba	
17	qmof-721171e		Zn_4O	edba	
18	qmof-79d5925		Zn_4O	edba	
19	Zn-H3BCTB	Zn-H3BCTB	Zn_4O	tcpb	
20	TOHYUM_clean		Zn_4O	ipa	
A	CAU21	CAU-21	Al_8O_8	func.	Type IV: Ten Al-based MOFs with building blocks similar to the selected ones. The organic ligands either extend the scaffolds of the seven selected MOFs (scaff.) or introduce functional groups (func.).
B	qmof-4b9877b		Al-O	scaff.	
C	qmof-6cf7eac		Al-O	scaff.	
D	qmof-26a5292		Al-OH	func.	
E	qmof-68edf8e		Al-OH	scaff. func.	
F	qmof-7044cf9		Al-OH	func.	
G	qmof-66998fd		Al-OH	func.	
H	qmof-ca29387		Al-OH	func.	
I	qmof-de9cc08		Al-OH	scaff. func.	
J	qmof-66998fd		Al_3O	scaff. func.	

Supplementary Table S1: List of MOFs used in this work.

In Table S1, we listed the MOFs used in this work and their building blocks.

3 Choice of MACE foundation model

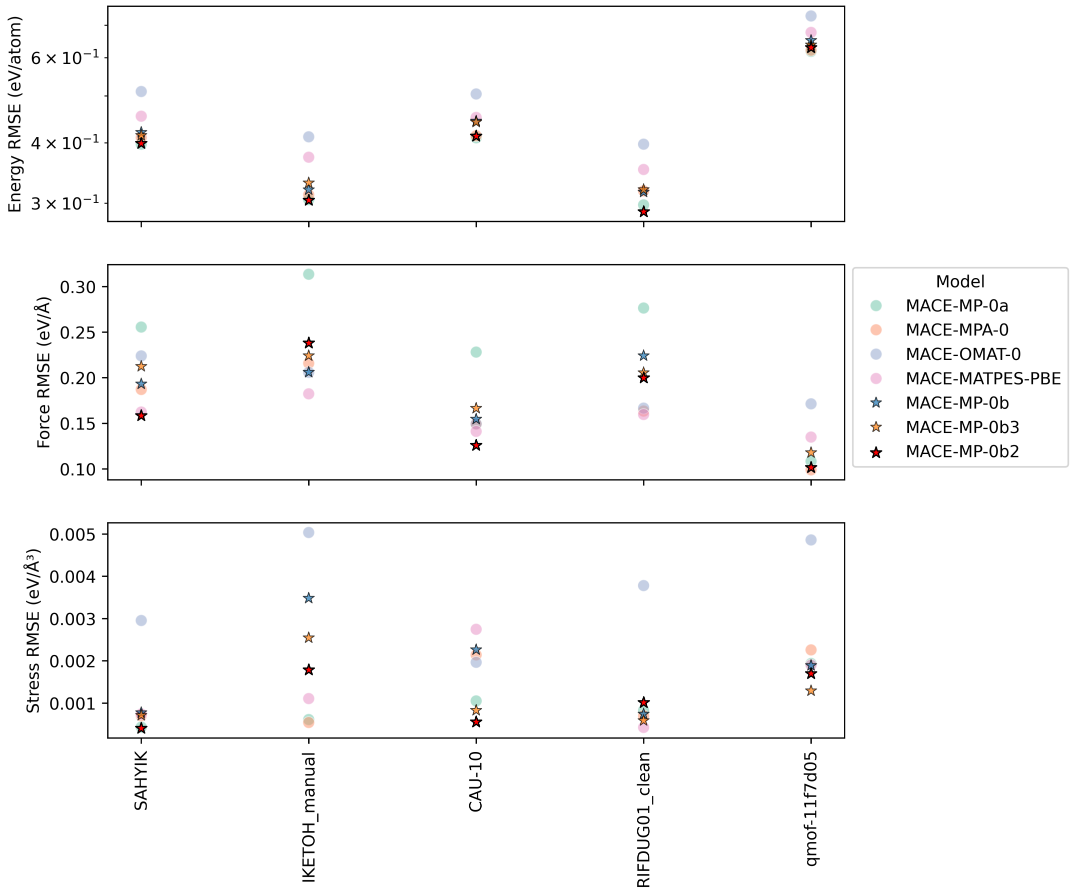

Supplementary Figure S2: Performance of MACE foundation models on the five MOFs of type II.

A series of MACE foundation models has been released, with the second-generation models (MACE-MP-0b*) recommended for fine-tuning due to their improved stability in MD simulations.[4] We initially selected MACE-MP-0b2, as it showed the best performance on CAU-10 among the second-generation models. The performance of medium-sized models on the five type-II MOFs is summarized in Fig. S2. None of the models achieves consistently superior performance across all systems.

4 The number of building blocks in training

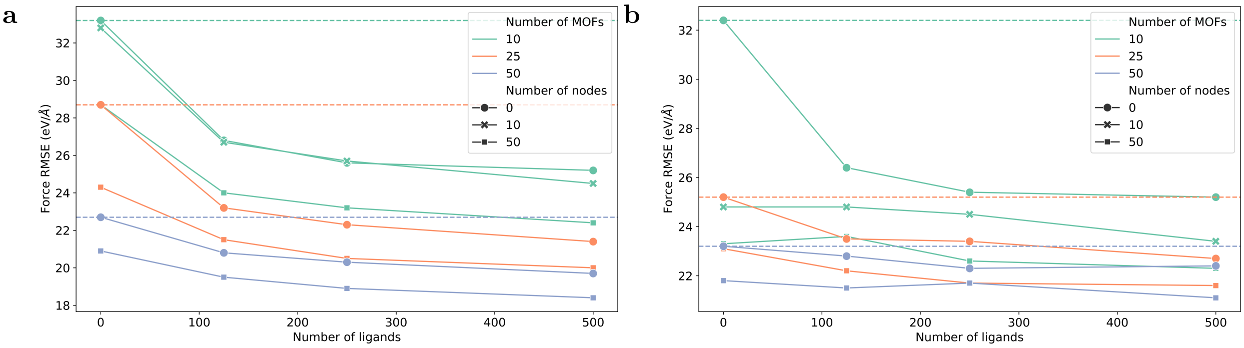

Supplementary Figure S3: Model performance with varying numbers of metal nodes and organic ligands for the training of (a) $Zn_4O(TCPB)_n$ and (b) MOF-14. The dashed lines represent the benchmark models, which were trained solely on frameworks.

In the $Zn_4O(TCPB)_n$ and MOF-14 cases discussed in Section 2.2 of the main text, we examined the effect of varying the number of metal nodes and organic ligands, as shown in Figs. S3a and S3b, respectively. Both figures reveal a consistent trend: model performance improves steadily as the number of building blocks in the training set increases.

5 Model performance on the twenty test MOFs

Figures S4 to S6 show parity plots comparing DFT reference values with machine-learning potential predictions for energy, force, and stress, respectively. Each subplot corresponds to one of the twenty MOFs that were excluded from training for MACE-FFLAME-N5L7.

In Fig. S4, a pronounced energy shift can be seen between MACE-MP-0b2 predictions and the DFT-calculated energies, even after baseline alignment. This systematic error arises from the lack of organic covalent bonding environments in the original MACE training set. Fine-tuning with ligand and MOF training data substantially reduces this shift in MACE-FFLAME-N5L7, though some structures (e.g., qmof-f80342c and TOHYUM_clean) still require additional refinement.

Force predictions from MACE-MP-0b2 show a relatively broader scatter as shown in Fig. S5. Fine-tuning with MACE-FFLAME-N5L7 significantly improves the accuracy, and a further round of fine-tuning with additional MOF data (MACE-FFLAME-MOF25) yields nearly perfect force predictions.

For stress predictions (Fig. S6), MACE-MP-0b2 tends to both overestimate and underestimate relative to DFT. MACE-FFLAME-N5L7 effectively corrects most of these deviations, and MACE-FFLAME-MOF25 further reduces the residual errors.

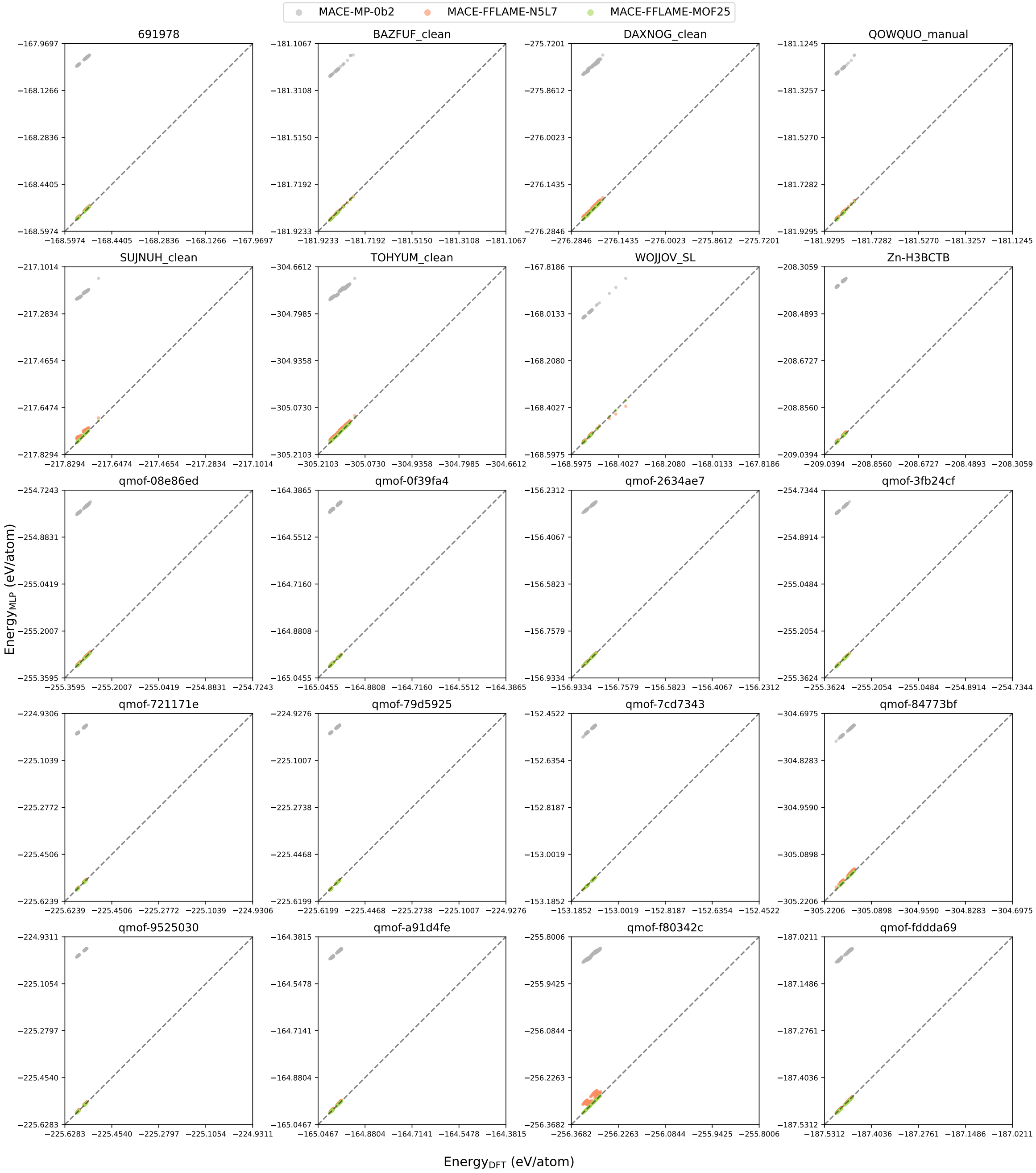

Supplementary Figure S4: Parity plots of DFT-calculated vs. predicted energies for the twenty test MOFs. Each subplot corresponds to an MOF of type III.

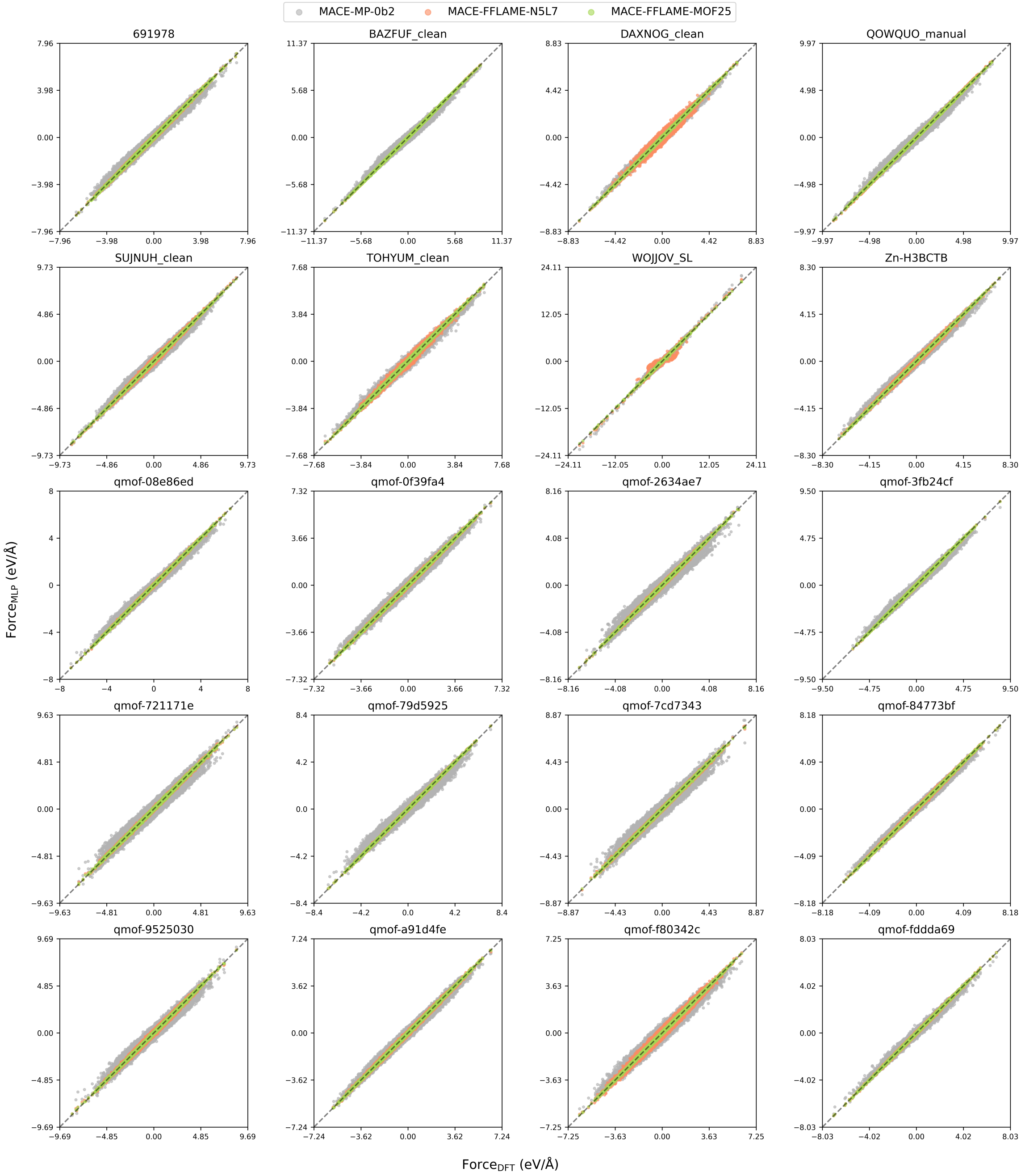

Supplementary Figure S5: Parity plots of DFT-calculated vs. predicted forces for the twenty test MOFs. Each subplot corresponds to an MOF of type III.

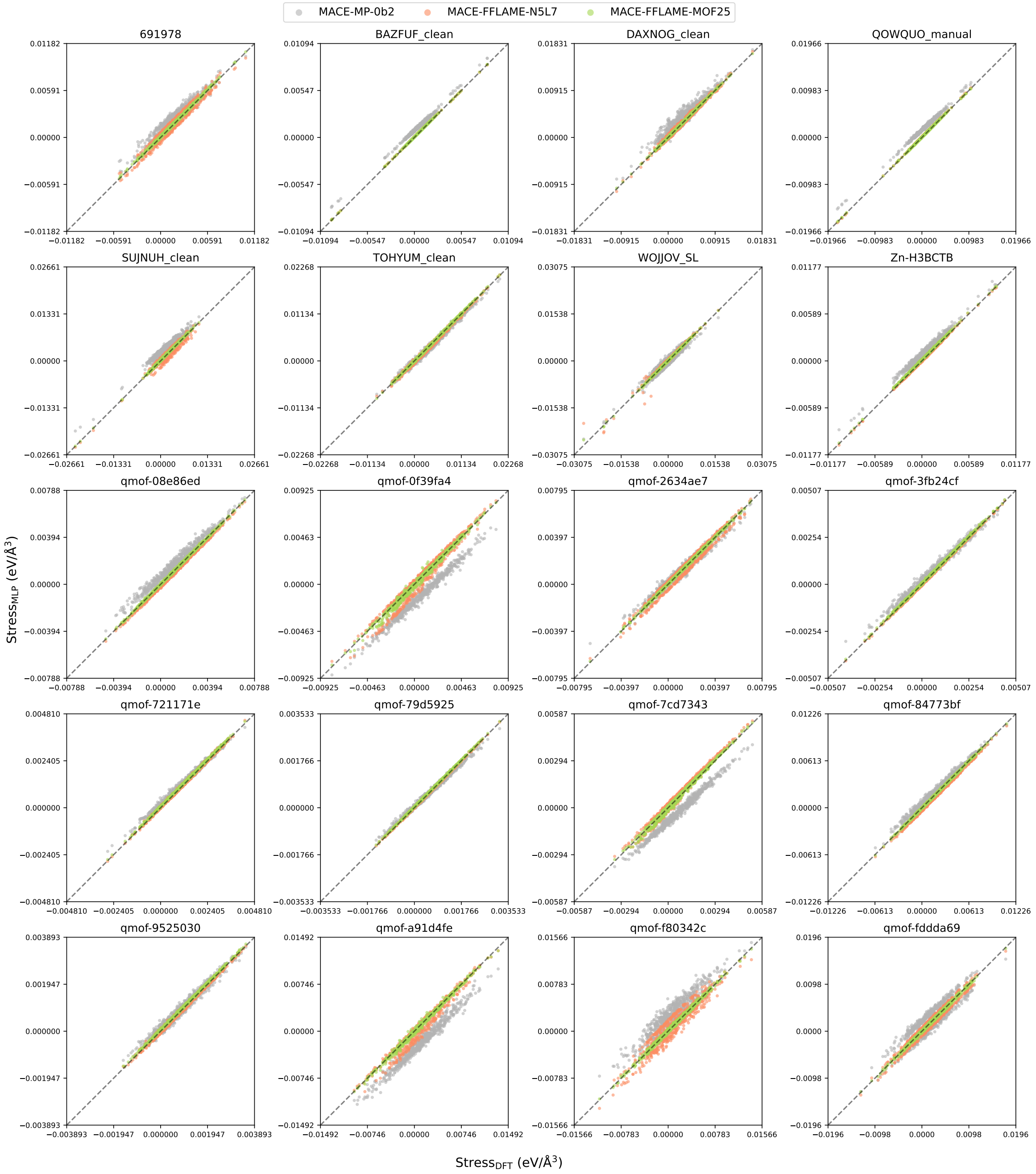

Supplementary Figure S6: Parity plots of DFT-calculated vs. predicted stresses for the twenty test MOFs. Each subplot corresponds to an MOF of type III.

6 Model performance on the five MOFs of type II

Figure S7 presents the model performance on the five MOFs used to sample node configurations. Our model, MACE-`FFLAME`-N5L7, exhibits significantly lower errors in energies, forces, and stresses compared to the original MACE model. After an additional round of fine-tuning on the remaining 20 MOFs not included in the original training set, the extended model, MACE-`FFLAME`-MOF25, continues to demonstrate excellent performance on these five benchmark MOFs, indicating strong generalization capabilities.

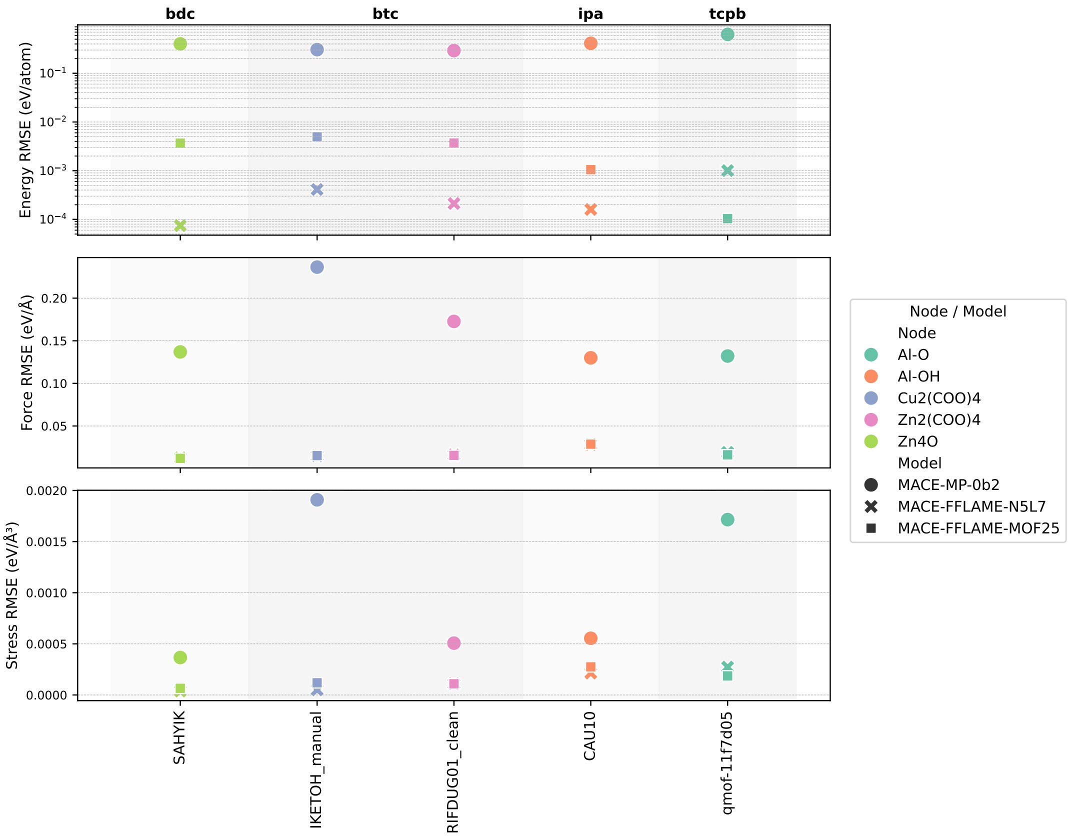

Supplementary Figure S7: Comparison of model errors (energies, forces, and stresses) on the five MOFs of type II. Both fine-tuned models show significantly improved accuracy over the original MACE baseline.

7 Force and stress errors on MOF-5

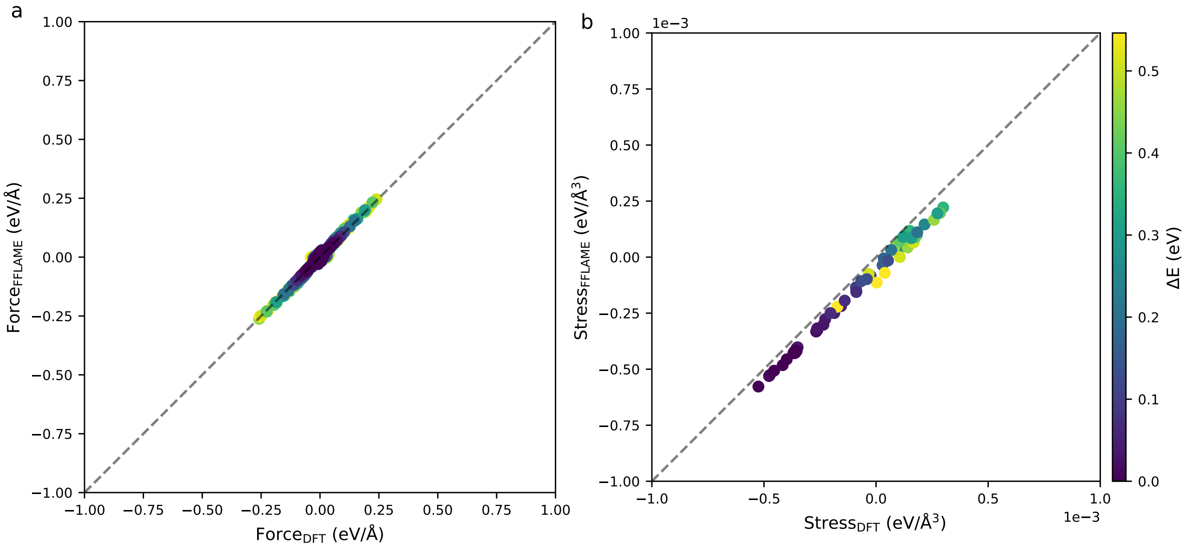

Supplementary Figure S8: Comparison of DFT and MACE-`FFLAME`-N5L7 predictions for the forces and stresses of MOF-5 configurations. The color of the points indicates the relative energy with respect to the minimum-energy configuration.

In Fig. S8, we compare the forces and stresses obtained from DFT and those predicted by MACE-`FFLAME`-N5L7 for the MOF-5 configurations employed in evaluating the rotational energy barrier of the phenylene group. The color of each point represents the relative energy of the corresponding configuration. Overall, the MACE-`FFLAME`-N5L7 model reproduces the DFT-calculated forces and stresses with excellent agreement, indicating its strong reliability and transferability across different configurations.

8 Faster training convergence

Here, we focus on fine-tuning a model specifically for MIL-53. The complete training configurations are taken from the work of Vandenhaute et al.[5]. We relabeled all configurations using the DFT functionals described in the Methods section of the main text and selected training subsets via K-means clustering, as also detailed there.

Training subsets of 300, 500, 700, and 900 representative configurations were constructed. Each subset was used to fine-tune both MACE and our MACE-`FFLAME`-N5L7 models.

All subsets lie within the data-rich regime, ensuring that the fine-tuned models successfully capture the breathing behavior of MIL-53 in molecular dynamics simulations.

Figure S9 presents the validation loss throughout the training process. The sharp drop in the loss curves corresponds to the two-stage training strategy employed. The legend indicates the foundation model and the number of training data points used. For each configuration, we performed four fine-tuning runs with different random seeds and plotted the one that converged most rapidly.

When fine-tuning from our `FFLAME` model, the initial loss is lower and convergence is achieved more quickly compared to MACE, resulting in over 50% reduction in GPU time.

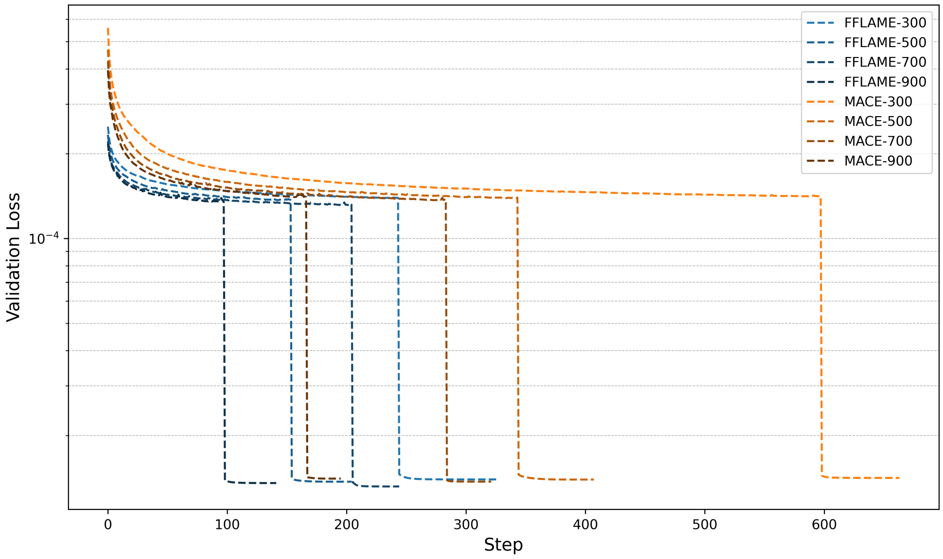

Supplementary Figure S9: Validation loss during fine-tuning under a data-rich setting. Each curve corresponds to the fastest-converging run (out of four seeds) for a given number of training configurations (300, 500, 700, or 900, as indicated in the legend). Fine-tuning from `FFLAME` leads to faster convergence than from MACE, reducing GPU time by over 50%.

References

[1] Yongchul G Chung, Emmanuel Haldoupis, Benjamin J Bucior, Maciej Haranczyk, Seulchan Lee, Hongda Zhang, Konstantinos D Vogiatzis, Marija Milisavljevic, Sanliang Ling, Jeffrey S Camp, et al. Advances, updates, and analytics for the computation-ready, experimental metal–organic framework database: Core mof 2019. *Journal of Chemical & Engineering Data*, 64(12):5985–5998, 2019.

[2] Andrew S Rosen, Shaelyn M Iyer, Debmalya Ray, Zhenpeng Yao, Alán Aspuru-Guzik, Laura Gagliardi, Justin M Notestein, and Randall Q Snurr. Machine learning the quantum-chemical properties of metal–organic frameworks for accelerated materials discovery. *Matter*, 4(5):1578–1597, 2021.

[3] Andrew S Rosen, Victor Fung, Patrick Huck, Cody T ODonnell, Matthew K Horton, Donald G Truhlar, Kristin A Persson, Justin M Notestein, and Randall Q Snurr. High-throughput predictions of metal–organic framework electronic properties: theoretical challenges, graph neural networks, and data exploration. *npj Computational Materials*, 8(1):112, 2022.

[4] Ilyes Batatia, Philipp Benner, Yuan Chiang, Alin M. Elena, Dávid P. Kovács, Janosh Riebesell, Xavier R. Advincula, Mark Asta, William J. Baldwin, Noam Bernstein, Arghya Bhowmik, Samuel M. Blau, Vlad Crare, James P. Darby, Sandip De, Flaviano Della Pia, Volker L. Deringer, Rokas Elijoius, Zakariya El-Machachi, Edvin Fako, Andrea C. Ferrari, Annalena Genreith-Schriever, Janine George, Rhys E. A. Goodall, Clare P. Grey, Shuang Han, Will Handley, Hendrik H. Heenen, Kersti Hermansson, Christian Holm, Jad Jaafar, Stephan Hofmann, Konstantin S. Jakob, Hyunwook Jung, Venkat Kapil, Aaron D. Kaplan, Nima Karimitari, Namu Kroupa, Jolla Kullgren, Matthew C. Kuner, Domantas Kuryla, Guoda Liepuoniute, Johannes T. Margraf, Ioan-Bogdan Magdu, Angelos Michaelides, J. Harry Moore, Aakash A. Naik, Samuel P. Niblett, Sam Walton Norwood, Niamh O'Neill, Christoph Ortner, Kristin A. Persson, Karsten Reuter, Andrew S. Rosen, Lars L. Schaaf, Christoph Schran, Eric Sivonxay, Tamás K.

Stenczel, Viktor Svahn, Christopher Sutton, Cas van der Oord, Eszter Varga-Umbrich, Tejs Vegge, Martin Vondrák, Yangshuai Wang, William C. Witt, Fabian Zills, and Gábor Csányi. A foundation model for atomistic materials chemistry. *arXiv preprint*, 2023. URL https://github.com/ACEsuit/mace-foundations/releases/download/mace_mp_0b2/mace-medium-density-agnesi-stress.model.

[5] Sander Vandenhaute, Maarten Cools-Ceuppens, Simon DeKeyser, Toon Verstraelen, and Veronique Van Speybroeck. Machine learning potentials for metal-organic frameworks using an incremental learning approach. *npj Computational Materials*, 9(1):19, 2023.
